# Mental distress predicts divorce over 16 years: the HUNT study

**DOI:** 10.1186/s12889-015-1662-0

**Published:** 2015-04-01

**Authors:** Mariann Idstad, Fartein Ask Torvik, Ingrid Borren, Kamilla Rognmo, Espen Røysamb, Kristian Tambs

**Affiliations:** Division of Mental Health, Norwegian Institute of Public Health, PO Box 4404 Nydalen N-0403, Oslo, Norway; University of Oslo, Oslo, Norway

## Abstract

**Background:**

The association between mental distress and divorce is well established in the literature. Explanations are commonly classified within two different frameworks; social selection (mentally distressed people are selected out of marriage) and social causation (divorce causes mental distress). Despite a relatively large body of literature on this subject, selection effects are somewhat less studied, and research based on data from both spouses is scarce. The purpose of the present study is to investigate selection effects both at the individual level and the couple level.

**Methods:**

The current study is based on couple-level data from a Norwegian representative sample including 20,233 couples. Long-term selection effects were tested for by means of Cox proportional hazard models, using mental distress in both partners at baseline as predictors of divorce the next 16 years. Three identical sets of analyses were run. The first included the total sample, whereas the second and third excluded couples who divorced within the first 4 or 8 years after baseline, respectively. An interaction term between mental distress in husband and in wife was specified and tested.

**Results:**

Hazard of divorce was significantly higher in couples with one mentally distressed partner than in couples with no mental distress in all analyses. There was also a significant interaction effect showing that the hazard of divorce for couples with two mentally distressed partners was higher than for couples with one mentally distressed partner, but lower than what could be expected from the combined main effects of two mentally distressed partners.

**Conclusions:**

Our results suggest that mentally distressed individuals are selected out of marriage. We also found support for a couple-level effect in which spouse similarity in mental distress to a certain degree seems to protect against divorce.

**Electronic supplementary material:**

The online version of this article (doi:10.1186/s12889-015-1662-0) contains supplementary material, which is available to authorized users.

## Background

There are several potential detrimental effects of divorce, such as lowered well-being, financial problems, long-term impaired functioning [1], and an elevated risk of mortality [2].

Mental distress and/or mental illness are important factors associated with divorce [3-10]. Explanations for this association are commonly classified within two different frameworks; social selection and social causation [11]. In social selection it is assumed that mental distress leads to divorce and that mentally distressed people are thus “selected” out of marriage. Social causation posits that divorce leads to mental distress due to the stresses of the transition into divorce and the new status as divorcee.

Although there is broad support in the literature for the social causation hypothesis, selection effects may occur simultaneously [1]. In fact, several studies have found support for both social causation and social selection [12-14]. For example, in a longitudinal study based on three-wave panel data from 930 respondents, Mastekaasa [15] reported evidence for a short-term selection effect as well as a long-term social causation effect.

The present study focuses on social selection. There are a number of different pathways from mental distress and mental illness to divorce. Mental disorders may negatively affect people’s ability to maintain marital relationships [5], which may in turn lead to divorce. For example, depressed patients and even patients with depressive symptoms tend to have impaired functioning both physically and socially, experience bodily pain and spend as many days in bed as people with chronic medical conditions [16]. Mental illness is also related to low levels of social capital [17]. Consequently, this may reduce mentally ill people’s capacity to participate in joint activities and provide their spouse with emotional support, which is important for companionship and individual well-being in spouses [18]. Another pathway from mental distress to divorce may lead through socioeconomic status. Mental illness is associated with low education, unemployment [19] and low family income [20]. Since level of education, receiving welfare and level of income are also related to marital quality and (in) stability [21], socioeconomic status may play a role in divorce. A third approach concerns similarities between partners. Research has consistently shown that partners resemble each other on mental and physical health as well as health behaviours [22]. Explanations for this have been categorized into two main categories: non-random mating on the one hand, which entails similarity in partners even before they meet, and, on the other hand, increased similarity due to shared experiences and mutual influence after partnering, such as emotional contagion [23]. Thus, people that are vulnerable and/or predisposed to develop mental distress may tend to select each other as partners, and may be exposed to the same negative life events or to mutual influence. This may in turn increase the risk of divorce, since couples with two mentally distressed partners are at an especially high risk of getting divorced [7,24].

An important limitation in the literature is the lack of research based on data from both spouses [25]. Couple data are necessary in order to identify to what extent the association between mental distress and divorce exists at the individual level or at the couple level. Data on mental distress status of only one spouse is sufficient to examine whether people with mental distress get divorced more frequently, which is important in itself. However, data on mental distress status of both spouses give a unique possibility to take one step further than what has been possible in most previous studies. The risk in couples with two mentally distressed spouses could be exactly as calculated from combining the individual risk in each of the spouses (multiplying the excess risk in distressed wives with the excess risk in distressed husbands). However, there could also be an additional risk in doubly burdened couples not accounted for by the individual risks alone. For instance the distressed partners might be unable to care for the other. Or, on the contrary, the risk in doubly burdened couples could be lower than expected from combining the individual risks, perhaps because sharing the burden may give some comfort. Detecting such effects, in which the presence or absence of distress in one spouse moderates the risk of divorce associated with distress in the other spouse, requires data from both spouses in very large samples, like ours.

An early study based on couple data from a small, clinical sample reported that couples in which one partner was depressed did not have higher divorce rates than the general population, whereas couples in which both partners were depressed had a divorce rate 8 times higher, suggesting a couple-level effect in which the two partners did not have sufficient capacity to support and help each other [24]. Butterworth and Rodgers [7] recently tested the generalizability of this finding utilizing representative data, investigating whether the mental health problems of both spouses in 3,230 couples measured at baseline could predict marital dissolution during the subsequent 36 months. The results showed that couples with one or two partners with mental health problems were significantly more likely to separate/divorce than couples without mental health problems, but there were no significant interaction effect between mental health problems in husbands and wives. Although these results supported Merikangas’ [24] finding that couples with two depressed partners have high divorce rates, they did not support the notion of a couple-level phenomenon. Butterworth and Rodgers [7] thus concluded that their results seemed to reflect an additive effect of individual mental health problems rather than a couple-level mental health effect. Finally, the authors noted that although their results suggested a selection effect, the time frame of three years did not allow for an exclusion of social causation.

In sum, most previous research is based on information from one spouse only, and where information from both spouses were available, statistical power may have been insufficient despite relatively large samples. Hence, little is still known about couple-level selection effects.

The current study applies a longitudinal design attempting to replicate the findings of Mastekaasa [15] and Butterworth and Rodgers [7]. Our study expands on previous research in important ways. Whereas the abovementioned studies were based on 930 and 3,230 couples, respectively, our sample includes more than 20,000 couples, implying high statistical power. Unlike the relatively short three-year period in Butterworth and Rodgers’ study [7], our 16 years follow-up period makes it possible to draw firmer conclusions with respect to selection effects not confounded by causation effects. As opposed to the study by Mastekaasa [15], our data include information from both spouses, which allow us to test for combined main effects and/or interaction effects. To the best of our knowledge, this is the first prospective study based on a large, representative sample including data from both spouses to examine long-term selection effects. The aims of the study are as follows:

1. Examine the association between mental distress and divorce over time, testing for long-term selection effects.
2. Investigate the extent to which such effects reflect an individual-level or a couple-level phenomenon.

## Methods

### Sample

The present study is based on data from the first wave of the Nord-Trøndelag Health Study (HUNT 1) in Norway carried out in 1984-86. The participants are followed through registries until year 2000. All inhabitants in Nord-Trøndelag County aged 20 years and older were invited to participate in HUNT 1. The county population is relatively stable and homogeneous, and fairly representative of the Norwegian general population in terms of geography, economy, industry, sources of income, age distribution, morbidity and mortality, although less urban [26] and with somewhat lower education and income. Two questionnaires, Q1 and Q2, were administered, of which the first questionnaire was returned at the examination site, and the second was handed out during the examination and returned by pre-paid mail. Out of the total adult population of 85,125 persons, 90.7%, completed Q1 and 75.1% returned both Q1 and Q2. The samples and screening procedures are described in further detail elsewhere [27]. Statistics Norway used the personal identification number assigned to Norwegian citizens to identify registered couples. For the present analyses, only individuals from married couples in which both spouses had valid data on both Q1 and Q2 were selected, resulting in a sample of 20,233 mixed-sex couples. This corresponds to 70.8% of all the couples invited to participate in HUNT 1.

### Ethics

HUNT 1 was approved by the Norwegian Data Inspectorate and the Regional Committee for Medical Research Ethics. Approval to use the data was provided by the HUNT Research Centre.

### Measures

#### Divorce

The hazard function in our study pertains to whether or not a couple got separated or divorced, referred to as divorce (D). Information on D during the 16 years of follow-up (1984-86 to 2000) was provided by Statistics Norway.

#### Global mental health

The main predictors are husbands’ and wives’ scores on the Global Mental Health measure (GMH). The GMH was based on nine items from Q1 and Q2. The items are presented in Table 1 along with their response categories and psychometric properties. Due to the different response categories, the scores were standardized before a summative index was generated. These items have also been used in mental health indices in previous studies [28,29]. We weighted the nine mental health items before including them in a summative index in order to further ensure that the index actually measures mental health. To obtain the weights, we ran a multiple linear regression analysis in another available data set with 6,380 subjects [30]. This material included both the mentioned nine items and the Hopkins Symptom Check List-25 (SCL-25) [31], designed to measure symptoms of anxiety and depression. The logarithm-transformed SCL-25 score was regressed on the nine items, and the regression coefficients from this analysis were used as weights for the summative index, GMH. The correlation between GMH and logarithm-transformed SCL-25 was 0.83 (0.82 for women and 0.84 for men). Cronbach’s alpha for GMH was 0.80 for husbands and 0.84 for wives. A high score on the GMH indicates poor mental health. The GMH was dichotomized into a new mental distress variable, MD, recoding the top 10 percentile to ‘1’ and lower scores to ‘0’. The 10% prevalence corresponds quite well with regular prevalence estimates for depression. 1976 men and 2070 women were classified with MD, corresponding to a total of 4046 couples in which one or both spouses were classified with MD.

**Table 1:** **The Global Mental Health index (GMH)**

**Item**	**Response categories**	**r**	**β**	**Corrected item-total correlation**
Do you often feel lonely?	Very often	0.65	0.32	0.46
	Often			
	Sometimes			
	Very rarely			
	Never			
During the last month, have you suffered from nervousness (felt irritable, anxious, tense or restless)?	Almost all the time	0.85	0.30	0.59
	Often			
	Sometimes			
	Never			
Do you feel, for the most part, strong and fit or tired and worn out?	Very strong and fit	0.69	0.17	0.60
	Strong and fit			
	Somewhat strong and fit			
	Somewhere in between			
	Somewhat tired and worn out			
	Tired and worn out			
	Very tired and worn out			
During the last month, have you had any problems falling asleep or sleep disorders?	Almost every night	0.62	0.13	0.48
	Often			
	Sometimes			
	Never			
Do you suffer from any long-term illness or injury of a physical or psychological nature that impairs your functioning in your everyday life? (Long-term means that it has lasted or will last for at least one year.) If YES, would you describe your impairment as slight, moderate or severe? [Among different types of impairment] Impairment due to mental health problems	Slight	0.50	0.12	0.33
	Moderate			
	Severe			
Do you by and large feel calm and good?	Almost all the time	0.74	0.11	0.61
	Often			
	Sometimes			
	Never			
Thinking about your life at the moment, would you say that you are by and large satisfied with life, or that you are mostly dissatisfied with your life?	Very satisfied	0.60	0.03	0.56
	Satisfied			
	Somewhat satisfied			
	Neither satisfied nor			
	dissatisfied			
	Somewhat dissatisfied			
	Dissatisfied			
	Very dissatisfied			
Would you say you are usually cheerful or downhearted?	Very downhearted	0.60	0.02	0.56
	Downhearted			
	Somewhat downhearted			
	Some of both			
	Somewhat cheerful			
	Cheerful			
	Very cheerful			
How often have you taken tranquilizers/sedatives or sleep medication during the	Daily	0.50	0.01	0.41
	last month?			
	Weekly, but not every day			
	Not as often as every week			
	Never			

Response categories and psychometric properties: items’ correlation with (r) and relative contributions to (β) the GMH, and corrected item-total correlations.

#### Demographic variables and covariates

We adjusted for the following demographic variables: couples’ mean age, both spouses’ education, years of marriage, and whether they were living together with children 5 years or younger, children 6-15 years or children older than 15 years. Covariates included self-reported physical health, perceived social support and alcohol use in both spouses.

Living with children included three items: *Do you live alone or with others? Place a cross next to those you live with.* [Seven sub-items, among which were the following three:] Children under 5 years old; Children between 6 - 15 years; Children over 15 years.

Self-reported physical health was measured by one item: *How is your health at the moment?* (Poor/Not so good/Good/Verygood).

Perceived social support was measured by one item: *If you fell ill and had to stay in bed over an extended period of time, how probable is it that you could count on receiving the necessary help and support from family, friends or neighbours?* (Very probable/ Fairly probable/Doubtful/Unlikely/Highly unlikely). The variable was recoded so that a high score corresponded to a high probability.

Alcohol use was measured by two items: *How frequently have you drunk alcohol (beer, wine or spirits) during the LAST 14 DAYS?* (I am a total abstainer, I never drink alcohol/ I have not drunk alcohol, although I am not a total abstainer/I have drunk alcohol 1 - 4 times/I have drunk alcohol 5 - 10 times/I have drunk alcohol more than 10 times) coded 0-4, and *If you have drunk alcohol during the course of the past 14 days, did it result in your feeling intoxicated on any occasion?* (yes/no). The two items were both standardized and then computed into a summative index. The index was standardized before used in the analyses.

### Design and statistical analyses

The data were organized so that each observation, or record, in the data file represents a couple. Thus, the couple is the unit of analysis. A longitudinal design was applied, investigating the hazard of D (divorce) in couples from baseline in 1984-86 to year 2000. If one of the spouses died during that period the observation was registered as right censored. Dependent variable was time from examination year to year of D. Cox regression analyses were conducted with MD in couples (in husband and in wife separately) as the principal predictors, providing estimates of the effect of male and female MD on hazard of D after adjustment for demographic variables and other covariates. All demographic variables and covariates were entered for husbands and wives, respectively, except for years of marriage and children living at home, which were measured at the couple level and thus entered only once. MD was measured at baseline, and information on D was available for each year up to year 2000. Male and female MD were entered as factors with two categories; respondents with no MD, and respondents who were classified with MD. Finally, because an effect of one partner’s MD on a couple’s eventual D might vary with the other partner’s MD, an interaction term between MD in the husbands and MD in the wives was specified and tested. Three identical sets of cox regression analyses were run. The first analysis included all the 20,233 couples in our sample. Because this first analysis involved a risk of reversed causality, the second analysis was run excluding couples who divorced within 4 years after baseline, reducing the sample to 19,511 couples. The third analysis excluded couples who divorced within 8 years after baseline, further reducing the sample to 19,024 couples. The time intervals of 4 and 8 years were chosen in order to be able to compare our results with those from Mastekaasa’s study [17] (1995).

### Treatment of missing values

We used SPSS Missing Value Analysis (MVA), expectation maximization for imputation of missing values in respondents with valid data on at least half of the items within a measure. For the GMH index, the nine items were used as predictors for each other, reducing missing values (at least one item missing) from 10.4% to 1.5% for men and from 12.6% to 1.6% for women. For alcohol use, the two items were used as predictors for each other, after categorizing respondents who had reported to be non-drinkers or that they had not been drinking alcohol the last 14 days on the first item, as not feeling intoxicated on the second item. Missing values were reduced from 2.5% to 1.6% for men and from 3.8% to 2.8% for women. There were no missing values on the public registry based variables age and years of marriage. Missing data on education amounted to 3.7% of the men and 3.2% of the women and were replaced with the lowest level of education. For the items on living with children we used the wives’ data, missing values were coded “not living with children”. Only 0.2% of the men and 0.2% of the women did not answer the question about their physical health, and 3.0% of the men and 2.9% of the women did not report perceived social support. Missing values on these variables were replaced by the sample mean for men and women, respectively.

## Results

### Sample characteristics

Background characteristics of the sample are presented in Table 2. The correlation between husbands’ and wives’ GMH scores was 0.28, and the Spearman rank correlation between MD scores was 0.30. The distribution of MD and D in couples in the total sample is shown in Table 3. The table shows that the percentage of D is almost twice as high in couples with one mentally distressed partner as in couples with no MD, and the percentage is even higher for couples in which both partners suffer from MD. In all, 8.6% of all couples divorced. This means that in 29.6% of all couples who divorced one or both spouses suffered from MD.

**Table 2:** **Descriptive statistics**

**Variable**	**Mean**	**SD**	**Range**
Husbands’ general mental health	8.04	4.10	0-34 (High score = poor mental health)
Wives’ general mental health	8.88	4.53	0-34 (High score = poor mental health)
Husbands’ age	52.04	15.26	22-95
Wives’ age	48.85	15.00	21-101
Children under 5 years	0.18	0.38	0-1 (No, Yes)
Children 6 to 15 years	0.36	0.48	0-1 (No, Yes)
Children older than 15 years	0.38	0.47	0-1 (No, Yes)
Years of marriage	32.31	18.47	0-86
Husbands’ physical health	2.84	0.66	1-4 (Poor, Not so good, Good, Very good)
Wives’ physical health	2.85	0.66	1-4 (Poor, Not so good, Good, Very good)
Husbands’ alcohol use, frequency	1.60	0.85	0-4 (High score = high frequency)
Husbands’ alcohol use, felt intoxicated	0.23	0.42	0-1 (No, Yes)
Wives’ alcohol use, frequency	1.23	0.78	0-4 (High score = high frequency)
Wives’ alcohol use, felt intoxicated	0.07	0.25	0-1 (No, Yes)
Husbands’ social support	4.39	0.90	1-5 (high score = high level of support)
Wives’ social support	4.13	1.01	1-5 (high score = high level of support)

**Table 3:** **Distribution of mental distress (MD) and divorce in couples within each category**

**N Couples (% divorced)**		**Wives**		
		**No MD**	**MD**	**Total**
Husbands	No MD	16622 (7.4)	1635 (14.1)	18257 (8.0)
	MD	1541 (13.8)	435 (16.6)	1976 (14.4)
	Total	18163 (7.9)	2070 (14.6)	20233 (8.6)

### Effect of mental distress on hazard of divorce

Cox regression analyses were conducted with year until D as dependent variable and MD in husbands and wives, respectively, as the principal predictors. Analysis 1 tested the hazard of divorce in the total sample. Analysis 2 and 3 excluded couples who divorced within 4 and 8 years after baseline, respectively. This way, we can test whether the effect of mental distress on divorce is equally strong for couples who have been married for a long time (more than 4 years and more than 8 years, respectively). If the hazard remains strong throughout the observation period it is likely to reflect a selection effect. The unadjusted differences between the groups and the group differences adjusted for demographic variables and covariates are presented as hazard ratios (HR) in Table 4.

**Table 4:** **Mental distress (MD) in husbands and wives as predictors of divorce 1-16 years later**

	**Analysis 1 All couples included**	**Analysis 2 Excluding couples who divorced within 4 years after baseline**					**Analysis 3 Excluding couples who divorced within 8 years after baseline**		
**Model**	**HR**	**p**	**95% CI**	**HR**	**p**	**95% CI**	**HR**	**p**	**95% CI**
**Model 1: main effects**									
Husbands’ mental distress	1.83	.000	1.60–2.08	1.33	.005	1.09–1.62	1.34	.042	1.01–1.76
Wives’ mental distress	1.95	.000	1.72–2.21	1.61	.000	1.34–1.94	1.62	.000	1.25–2.10
**Model 2: main effects and interaction**									
Husbands’ mental distress	2.08	.000	1.79–2.41	1.54	.000	1.24–1.91	1.54	.005	1.14–2.09
Wives’ mental distress	2.19	.000	1.90–2.52	1.82	.000	1.50–2.21	1.83	.000	1.39–2.40
Interaction: Husbands’ MD by Wives’ MD	0.61	.002	0.45–0.83	0.49	.007	0.29–0.82	0.49	.059	0.24–1.03
**Model 3: main effects adjusted for demographic variables** ^1^ ^3^									
Husbands’ mental distress	2.07	.000	1.81–2.36	1.54	.000	1.26–1.87	1.58	.001	1.20–2.09
Wives’ mental distress	2.48	.000	2.19–2.82	2.15	.000	1.79–2.58	2.26	.000	1.74–2.92
**Model 4: main effects adjusted for demographic variables** ^1^ ^3^ **and covariates** ^2^ ^3^									
Husbands’ mental distress	1.90	.000	1.65–2.18	1.45	.001	1.17–1.78	1.51	.006	1.13–2.02
Wives’ mental distress	2.36	.000	2.05–2.71	1.96	.000	1.61–2.39	2.08	.000	1.57–2.74
**Model 5: main effects and interaction adjusted for demographic variables** ^1^ ^3^ **and covariates** ^2^ ^3^									
Husbands’ mental distress	2.10	.000	1.79–2.45	1.63	.000	1.30–2.04	1.69	.001	1.24–2.32
Wives’ mental distress	2.59	.000	2.22–3.02	2.17	.000	1.76–2.68	2.29	.000	1.70–3.07
Interaction: Husbands’ MD* Wives’ MD	0.67	.010	0.49–0.91	0.55	.024	0.32–0.92	0.56	.120	0.27–1.16

^1^Age, education, years of marriage, children living at home.

^2^Physical health, alcohol use, social support.

^3^All demographic variables and covariates were entered for husbands and wives, respectively, except for age, years of marriage and children living at home, which were measured at the couple level and entered only once.

Table 4, Analysis 1 shows the results from the analysis based on the total sample of 20,233 couples. The unadjusted results in Analysis 1 (models 1 and 2) show that couples in which the husband or the wife suffers from MD have about twice the hazard of D as couples without MD, and this effect remains strong after adjusting for demographic variables and covariates (models 3 and 4). The results for the interaction term show that the hazard rate for couples in which both partners suffer from MD is lower compared to what could be expected from the combined main effects, and this effect is valid throughout the analysis. The exact hazard ratios are obtained by multiplying the two main effects with the corresponding interaction effect, thus the hazard rate for couples in which both partners suffer from MD in Model 2 is 2.08*2.19*0.61 = 2.78, and the corresponding rate in Model 5 is 3.63. Combining the two main effects would correspond to a hazard rate of 2.08*2.19 = 4.55 in Model 2 and 5.41 in Model 5. This shows that the risk of D for couples with two mentally distressed partners is not as high as one might expect from the combined main effects, however, it is higher than the hazard for couples in which only one partner suffers from MD.

Table 4, Analysis 2 shows the results from the analysis in which couples who divorced within 4 years after baseline were excluded, N = 19,511. The unadjusted results in Analysis 2 (models 1 and 2) show that couples in which the husband or the wife suffers from MD have a higher hazard of D than couples without MD, ranging from 1.33 to 1.82, and this effect remains strong after adjusting for demographic variables and covariates. The value of the interaction term shows that the hazard rate for couples in which both partners suffer from MD is lower compared to what could be expected from the combined main effects, and this effect is valid throughout the analysis. The hazard ratios are 1.37 in Model 2 and 1.90 in Model 5. The combined main effects in the corresponding models would be 2.80 and 3.52. As in Analysis 1, the risk of D for couples with two mentally distressed partners is not as high as one might expect from the combined main effects. In Models 2 and 5 the risk for such couples is actually similar to the risk for couples in which only one partner suffers from MD. The results of all the included predictor variables from Analysis 2, Model 4 are shown in Additional file 1.

Table 4, Analysis 3 shows the results from the analysis in which couples who divorced within 8 years after baseline were excluded, N = 19,024. The unadjusted results in Analysis 3 (Models 1 and 2) are almost identical to the corresponding results reported in Analysis 2, showing that couples in which the husband or the wife suffers from MD have a higher hazard of D than couples without MD, ranging from 1.34 to 1.83, and this effect remains strong after adjusting for demographic variables and covariates. The interaction term was not significant in either model in this data set with a reduced statistical power. The estimates are highly similar to those from Analysis 2, however.

Finally, due to the relatively high numbers of missing on the GMH index, we reran the main analysis (Model 4, in Analysis 1, 2 and 3) with the original, unimputed GMH index to check the stability of the results. None of the parameter estimates changed with more than 0.05, and the p values remained very similar (with one p value changing from .006 to .001, and another one from .001 to <.0005). This shows that the results do not depend on the imputation of missing values.

## Discussion

The first aim of our study was to examine the association between mental distress and divorce over time, testing for long-term selection effects. In general, the results from the present study show that there is a significant association between mental distress and divorce. The current results expand on previous research by testing this association on longitudinal couple data from a large, representative sample. Couples with a mentally distressed husband or wife had more than a twofold risk of divorce compared to couples in which neither spouse suffered from mental distress, even after controlling for demographic variables and other covariates. The results show a peak in the effect of mental distress on hazard of divorce in the years immediately preceding the event and, further, that mental distress predicts divorce for as long as 8 years or more into the future. A social causation explanation for the peak in the effect of mental distress during the years around the divorce is plausible. However, the results showing a risk for divorce many years after the observation of mental distress provide evidence of a strong selection effect. This contradicts Mastekaasa’s [15] finding, but supports other longitudinal research [11,32,33].

Because marital problems have been found to predict divorce [34], one might argue that our results may reflect chronic problems within the marriage leading to mental distress and subsequent divorce, rather than mental distress being a direct cause. In fact, a recent review of couple- and family-based treatments in depression stated that there seems to be a reciprocal relationship between marital quality and depressive symptoms [35]. On the other hand, one study based on couple data found that although both partners’ degree of psychopathology was related to both partners’ degree of marital satisfaction, the more important factor for marital satisfaction was one’s own degree of psychopathology [36]. In other words, the poorer one’s mental health, the more dissatisfied one may be with one’s marriage. Thus, marital problems may also be a result of mental distress. Unfortunately, our data did not include information on marital satisfaction.

Like mental distress, alcohol use has been shown in the same data material to predict divorce [37], and alcohol use could well be suspected to mediate as well as confound the effect of mental distress. However, entering the demographic factors, social support, and life style, including alcohol use, as covariates did not change the estimates very much. These results imply that neither alcohol use nor the other covariates strongly confound or mediate the effects of mental distress on divorce.

The second aim of our study was to investigate whether the observed selection effects reflect an individual-level or couple-level phenomenon. To the extent that couples in our study concordant on mental distress have an especially high risk of divorce, our results support the findings of both Merikangas [24] and Butterworth and Rodgers [7]. However, our results show a significant interaction effect between husbands’ mental distress and wives’ mental distress in the first and second analyses, indicating that the elevated divorce risk among these couples is also related to mental distress at the couple-level. This contradicts the results from Butterworth and Rodgers’ study [7] which seemed to reflect only an additive effect of individual mental health problems, and no interaction effect between mental health in each of the spouses. The interaction effect in our study was no longer significant when excluding couples who divorced within 8 years after baseline, but this is likely to result from loss of statistical power. The number of couples in which both partners were mentally distressed and who also experienced divorce was reduced from 72 couples in the first analysis, to 20 couples in the second analysis, and to 10 couples in the final analysis, still the estimates in the final model were very similar to those in the previous model. Likewise, lack of power may explain why Butterworth and Rodgers [7] did not find evidence for an interaction effect.

Our finding of the interaction effect indicates that there may be a certain protective effect of being married to a person with a level of mental distress similar to ones own level, even in couples with two mentally distressed partners. This is supportive of the health mismatch hypothesis [38] which posits that couples with concordant health status are at a lower risk of getting divorced than couples with discordant health status. Similar findings have been reported in other research. In a recent study on alcohol use, concordant heavy drinking predicted divorce to a lesser extent than what was expected from the combined main effects, possibly due to perceived compatibility or a judgement that it may be difficult to deal with the problems alone and to find a new partner [37]. It is not difficult to imagine that this scenario might hold also for people with mental distress. Another explanation may be related to assortative mating, referring to the tendency for individuals to choose life partners with similar characteristics as themselves, which has been reported for psychiatric disorders [39]. It may be that people with similar mental health understand each other better, and are thus better able to cope with challenges related to couple mental distress. In conclusion, couples with one or two mentally distressed partners in our study have a persistently higher risk of divorce than couples in which neither partner suffers from mental distress. The divorce rate for couples with two mentally distressed partners was lower than expected, but still high. Thus, our results suggest that mentally distressed individuals are indeed selected out of marriage.

Although gender differences are not a focus of our study, we note that the effect of mental distress on divorce was stronger for women than for men, contrary to the finding in Butterworth and Rodgers’ study [7]. However, the sizes of the differences are well within what could be due to random fluctuations.

### Limitations

Unfortunately, our data did not include information on marital satisfaction. This is important, as the effect of women’s mental health problems on marital disruption disappeared when controlling for women’s relationship dissatisfaction in the study by Butterworth and Rodgers [7]. Likewise, Breslau and colleagues [5] noted that the observed relation between mental disorder and divorce across 12 countries in their study may have partly been a result of preceding marital distress.

Despite our large sample, lack of power is probably the reason why an interaction effect was not detected in our final analysis, since the estimates of the interaction effect is highly similar for all three sets of analyses.

The design of our study did not permit us to investigate both long-term social causation and social selection effects.

We do not know how well our results generalize to other societies. For instance the risk associated with mental illness in both spouses could be higher in a society in which mental health services are less available than in ours, and in which mentally ill spouses to a larger extent are left to take care of each other.

Another limitation pertains to the lack of information on whether some of the couples in our study were remarried, since people who have previously divorced are more likely to get divorced again. Furthermore, our sample included married couples only and not cohabiting couples. It is, however, unlikely that the inclusion of cohabiting couples would have represented a substantial change of the results. A study by Ask and colleagues [23] based on couple data from HUNT 1 estimated that about 1.2% of all the participating couples were cohabiting whereas the rest were married.

We chose to dichotomize our principal explanatory variables, mental distress in husband and wife. While this may be considered a limitation, because it implies losing some information, it makes the results more easily interpretable. Also, very skewed distributions of the MD variables make treating them as continuous predictor variables a little problematic.

Finally, despite our efforts to control for a wide range of covariates, we do not have information about circumstances that may occur in the period from baseline to year of divorce, such as the birth of (more) children, the death of relatives, changes in social support, changes in socioeconomic status, fluctuations in mental health and so on. Thus, our results should be interpreted with caution. For example, a major negative life event such as losing one’s job may negatively affect a family’s socioeconomic status and also the climate in a couple’s relationship and contribute to an eventual divorce.

However, our study has several strengths. It is based on couple data from a large, population based sample followed for many years. We were able to control for a range of relevant variables, including years of marriage, which is important because of higher dissolution rates in the earlier years of marriage [40].

Future studies should be based on data from both spouses and, ideally, follow people for some years before they marry and then for many years after, in order to be able to examine social causation as well as social selection in the same sample. It is not surprising that divorce may lead to mental distress, but the question of whether mentally distressed people are selected out of marriage may be less straightforward. In our study, mental distress apparently seems to lead to divorce, but this association may also be due to unknown third factors such as marital dissatisfaction, economic hardship or shared negative life events. Such factors should be studied in more detail. The dynamics of the shared climate of two mentally distressed spouses is also a subject that deserves more attention. Why is it that such couples in our study did not divorce as frequently as would have been expected from the double risk? Is it because both spouses lack the resources to implement the process of divorce, or have some of these couples developed certain strategies that help them understand each other and lead a relatively well functioning life together despite it all? Or maybe shared exposure to a major negative life event caused mental distress in both spouses but tied them closer together rather than result in marital conflicts. Answers to these questions may aid mental health professionals in identifying couples at risk for divorce, and in helping such couples to understand and deal with challenges related to mental distress both as individuals and couples.

## Conclusions

The current results make a new contribution to the literature, supplementing the very few studies in the field based on data from both partners. We found evidence for a selection effect, but there is still a need for research based on couple data in order to make assumptions on the dynamics of mental distress and divorce.

## Additional file

Additional file 1:
**Mental distress (MD) as predictor for divorce.**
