# Supplementary material for: Mental distress predicts divorce over 16 years: the HUNT study
Source: BMC Public Health. 2015 Apr 1;15:320. doi: 10.1186/s12889-015-1662-0 (PMC4394420; doi:10.1186/s12889-015-1662-0)
Supplement: Additional file 1: — Mental distress (MD) as predictor for divorce. [file 12889_2015_1662_MOESM1_ESM.docx]

Appendix I. Mental distress (MD) as predictor for divorce.

|  | **Analysis 2**  Excluding couples who divorced up to 4 years after baseline | | | |  |
| --- | --- | --- | --- | --- | --- |
| **Model** | **HR** | **p** | | **95% CI** | |
|  |  | |  |  | |
| **Model 4: main effects adjusted for background variables**¹  **and covariates**² |  | |  |  | |
| Husbands’ mental distress | 1.45 | | .001 | 1.17–1.78 | |
| Wives’ mental distress | 1.96 | | .000 | 1.61–2.39 | |
| Age | 0.90 | | .000 | 0.89-0.91 | |
| Husbands’ education (primary school) |  | | .408 |  | |
| Husbands’ education (secondary school) | 1.10 | | .184 | 0.96–1.28 | |
| Husbands’ education (university/college) | 1.03 | | .757 | 0.84–1.26 | |
| Wives’ education (primary school) |  | | .761 |  | |
| Wives’ education (secondary school) | 1.05 | | .487 | 0.91–1.22 | |
| Wives’ education (university/college) | 0.99 | | .956 | 0.79–1.25 | |
| Children under 5 years | 1.42 | | .000 | 1.22–1.66 | |
| Children 6 to 15 years | 0.80 | | .001 | 0.70–0.91 | |
| Children older than 15 years | 0.96 | | .658 | 0.81–1.14 | |
| Years of marriage | 1.01 | | .000 | 1.01-1.02 | |
| Husbands’ physical health | 0.96 | | .491 | 0.86–1.08 | |
| Wives’ physical health | 0.95 | | .373 | 0.85–1.06 | |
| Husbands’ alcohol use | 1.05 | | .160 | 0.98–1.12 | |
| Wives’ alcohol use | 1.06 | | .034 | 1.01–1.12 | |
| Husbands’ social support | 0.91 | | .033 | 0.84–0.99 | |
| Wives’ social support | 0.95 | | .175 | 0.88–1.02 | |

¹ Age, education, children living at home, years of marriage

² Physical health, alcohol use, social support
